# Enzymatic Modification of Flavonoids: Implications for Bioavailability, Bioactivity, and Therapeutic Potential Based on a Systematic Review and Bioinformatics Analysis

**DOI:** 10.3390/antiox15050539

**Published:** 2026-04-24

**Authors:** Marília Crivelari da Cunha, Mariana Crivelari da Cunha, Yasmin Bedani Scavone Ferrari, Nicolly Clemente de Melo, Lucas Miguel de Carvalho, Manoela Marques Ortega, Patrícia de Oliveira Carvalho

**Affiliations:** 1 Health Sciences Postgraduate Program, Universidade São Francisco (USF), Bragança Paulista 12916-900, SP, Brazil; marilia.crivelari@mail.usf.edu.br (M.C.d.C.); yasmin.ferrari@mail.usf.edu.br (Y.B.S.F.); nicolly.melo@mail.usf.edu.br (N.C.d.M.); lucas.miguel@usf.edu.br (L.M.d.C.); manoela.ortega@usf.edu.br (M.M.O.); 2 Undergraduate Program in Nutrition, Universidade São Francisco (USF), Itatiba 13251-900, SP, Brazil; mariana.crivelari@mail.usf.edu.br

**Keywords:** biocatalysis, bioavailability, structure–activity relationship, pharmacokinetics, clinical evidence

## Abstract

Flavonoids are a broad class of polyphenolic compounds with well-established biological and therapeutic relevance; however, their clinical application is often limited by unfavorable pharmacokinetic properties, including low solubility, poor bioavailability, and extensive metabolism. Enzymatic modification has emerged as a promising strategy to overcome these limitations, enabling regio- and stereoselective structural changes under mild and environmentally sustainable conditions. This study presents a systematic review conducted in accordance with the PRISMA 2020 guidelines, covering studies published between 2015 and 2025 on the enzymatic modification of flavonoids and their effects on physicochemical properties and bioavailability, with emphasis on biological activity and therapeutic potential. The literature search was performed in PubMed, Scopus, and Web of Science, complemented by citation searching and exploratory bioinformatics analysis. Evidence from in vitro, in vivo, and clinical studies indicates that enzymatic modifications such as glycosylation, deglycosylation, acylation, hydroxylation, methylation, and prenylation can improve stability, absorption, and biological activity. Despite promising results in experimental models, clinical evidence remains limited. Overall, enzymatic modification represents a promising strategy for improving flavonoid properties; however, most available evidence is derived from in vitro and in vivo studies, and further well-designed clinical trials are required to confirm their therapeutic potential in humans.

## 1. Introduction

Flavonoids constitute a broad class of polyphenolic compounds naturally occurring in plants, including fruits, vegetables, cereals, seeds, flowers, leaves, and bark [1,2]. These secondary metabolites are widely recognized for their diverse biological activities, including antioxidant, anti-inflammatory, antimicrobial, cardioprotective, and anticancer effects [1,2,3,4].

Despite these promising effects, the therapeutic application of flavonoids is constrained by pharmacokinetic factors, including their hydrophobic nature, reduced intestinal permeability, extensive first-pass metabolism, and rapid clearance, which result in limited systemic bioavailability [1]. Interactions with the food matrix and the gut microbiota significantly influence their absorption and metabolism, leading to variability in biological responses [5,6].

In plants, flavonoids predominantly occur as glycosides conjugated to different sugars, whose absorption in the gastrointestinal tract requires prior release of the more permeable aglycones; however, this process is often incomplete and variable, and is further exacerbated by extensive intestinal and hepatic metabolism, which limits the plasma concentration of active compounds [1].

Structural modification strategies aim to overcome these limitations, with particular emphasis on enzymatic approaches, which enable regio- and stereoselective transformations under mild conditions and with a lower environmental impact than conventional chemical routes [7]. Processes such as glycosylation/deglycosylation [8], acylation [9], hydroxylation [10], methylation [11], and prenylation [12] modify solubility, stability, and bioavailability, thereby improving or complementing physicochemical and biological properties [10,12,13,14,15].

Beyond altering physicochemical properties, these modifications enhance biological effects, including antioxidant [9,16], anti-inflammatory [17,18], antimicrobial [19], and anticancer activities [20]. The generation of novel structural derivatives enables the optimization of potency, selectivity, and pharmacological profiles, thereby expanding the potential applications of flavonoid-based bioactive compounds in pharmaceutical, nutraceutical, and functional food systems. However, these effects are highly context-dependent and may vary according to structural features, experimental models, and biological systems.

Due to the scarcity of reviews integrating the physicochemical and biological impacts of enzymatic approaches, especially those involving commercial and recombinant enzymes, the present review systematically compiles and critically analyzes recent literature (2015–2025) on the enzymatic biotransformation of flavonoids. The review identifies key advances, limitations, and challenges for future clinical translation, with particular emphasis on how these modifications influence physicochemical properties and bioavailability, and how these changes are associated with biological activity and therapeutic potential. The review is structured as a systematic analysis of experimental studies (in vitro and in vivo), including available clinical studies, and is supplemented by an exploratory bioinformatics analysis to contextualize flavonoid–disease associations reported in public databases.

## 2. Materials and Methods

The systematic review was conducted in accordance with PRISMA 2020 guidelines [21], and the PRISMA checklist was completed and provided to the journal during submission. The review protocol was not prospectively registered. The literature search was conducted in the PubMed, Scopus, and Web of Science databases. The last search was performed in January 2026. The search was supplemented by citation searching and manual searches on publisher platforms, including ScienceDirect, MDPI, and Springer Nature.

The search strategy combined keywords and Boolean operators (AND, OR) related to enzymatic modification of flavonoids, including terms such as “flavonoid”, “enzymatic modification”, “biocatalysis”, “glycosylation”, “deglycosylation”, “acylation”, “hydroxylation”, “methylation”, and “prenylation”, as well as terms associated with biological evaluation, such as “bioavailability”, “antioxidant”, “antimicrobial”, “anti-inflammatory”, and “anticancer”. The search terms were adapted as necessary for each database. Only articles published between 2015 and 2025 in English were considered. The full search strategy for each database is provided in the Supplementary Materials (Supplementary File S2).

Studies were included if they investigated the enzymatic modification of flavonoids and reported experimental data on physicochemical or biological properties, including in vitro, in vivo, or clinical models. Review articles, book chapters, conference abstracts, and studies that did not clearly describe the enzymatic process or did not include biological or physicochemical evaluation were excluded.

Study selection was performed by one reviewer and independently checked by a second reviewer. Titles and abstracts were screened according to the eligibility criteria, followed by full-text assessment of potentially relevant studies. Any disagreements regarding study eligibility were resolved through discussion until consensus was reached.

Data extraction was performed by one reviewer using a standardized data collection form and independently checked by a second reviewer to ensure accuracy and consistency. The extracted data included author and year of publication, type of enzymatic modification, flavonoid substrate, obtained derivatives, enzymatic systems, experimental model (in vitro, in vivo, or clinical), evaluated biological activity, and main outcomes. Additional data extracted included study design and, when available, information on funding sources. When information was missing or unclear, the study was included only if the enzymatic modification process and the biological outcomes were clearly described; otherwise, the study was excluded.

Due to the heterogeneity of experimental models, enzymatic systems, and outcome measures, a meta-analysis was not feasible; therefore, the results were synthesized qualitatively using a narrative approach. A formal risk-of-bias assessment using standardized tools was not applied due to the diversity of study designs, including in vitro, in vivo, and clinical studies. Nevertheless, methodological quality was assessed qualitatively considering the experimental model, presence of control groups, clarity of the enzymatic process, outcome measures, and reproducibility of the results, which contributed to the critical interpretation of the included studies.

The included studies were grouped according to the type of enzymatic modification (glycosylation, deglycosylation, acylation, hydroxylation, methylation, and prenylation), the biological activity evaluated, and the experimental model (in vitro, in vivo, or clinical).

**Figure 1:**
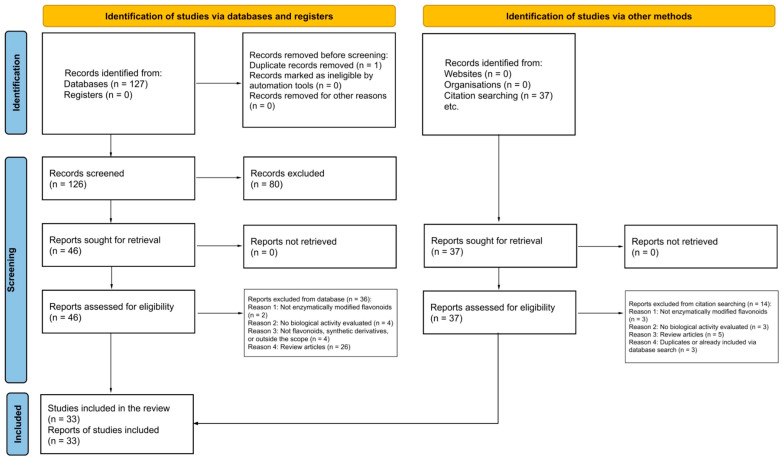
PRISMA flow diagram illustrating the stages of study identification, screening, and study inclusion in the literature review [23].

A total of 164 records were identified, including 127 from databases and 37 obtained through citation searching. After screening and eligibility assessment, 33 studies met the inclusion criteria and were included in the final analysis. The study selection process is presented in a flow diagram (Figure 1), and the PRISMA 2020 checklist is provided in the Supplementary Materials (Supplementary File S1). Studies that appeared to meet the inclusion criteria but were excluded after full-text assessment are listed in the Supplementary Materials (Supplementary File S2), along with the reasons for exclusion.

**Figure 2:**
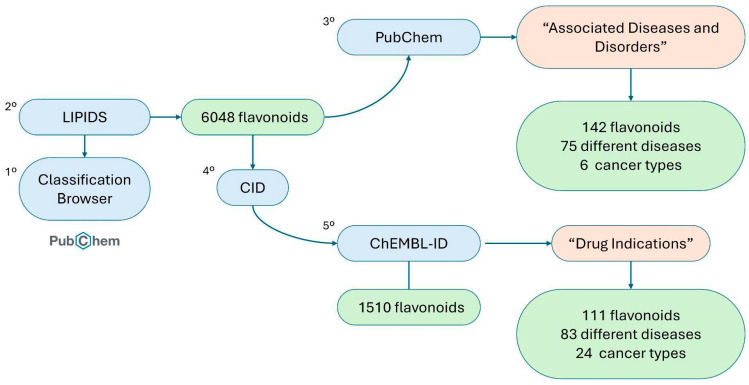
Flowchart of the bioinformatics-based analysis of flavonoid–disease associations. Author-generated illustration.

An exploratory bioinformatics analysis was conducted using public databases to complement the systematic review. This analysis was performed independently from the PRISMA-based systematic review and served solely to provide additional information on flavonoid–disease associations. The PubChem database, together with the LIPID MAPS classification system, was used to provide a broader perspective on potential disease associations of flavonoids, particularly with respect to cancer. PubChem, which compiles chemical, biological, and pharmacological information on compounds, was used to identify compound–disease associations. Compound selection was guided by the LIPID MAPS classification system, focusing on the Polyketides [PK12] category, which corresponds to flavonoids. Each entry is assigned a CID code, facilitating identification and cross-referencing within the database [22]. The overall workflow of this bioinformatics approach is summarized in Figure 2.

Data extraction and processing were conducted using Python-based automated queries (version 3.12.4) targeting the “Associated Diseases and Disorders” section of PubChem, which enabled the compilation of reported compound–disease associations. The extracted data were then organized and filtered to standardize terminology and eliminate duplicates, resulting in a structured dataset suitable for descriptive and categorical analysis.

The initial search targeted cancer-related associations using terms such as “neoplasm,” “tumor,” “metastatic,” “metastasis,” “leukemia,” “carcinoma,” and “sarcoma.” Due to the limited number of results, the search was broadened to include all diseases associated with flavonoid compounds. The associations reported in PubChem may reflect various contexts, including causal relationships, therapeutic effects, or biomarker associations (e.g., glucose and diabetes) [24].

The dataset was also cross-referenced with the ChEMBL platform using the corresponding CIDs to identify compounds with reported drug indications, particularly in the context of neoplastic disorders [25]. This exploratory bioinformatics analysis was conducted solely for contextual and descriptive purposes and was not included in the PRISMA eligibility or study selection process.

## 3. Flavonoids: Chemical Structure, Subclasses, and Biological Relevance

**Figure 3:**
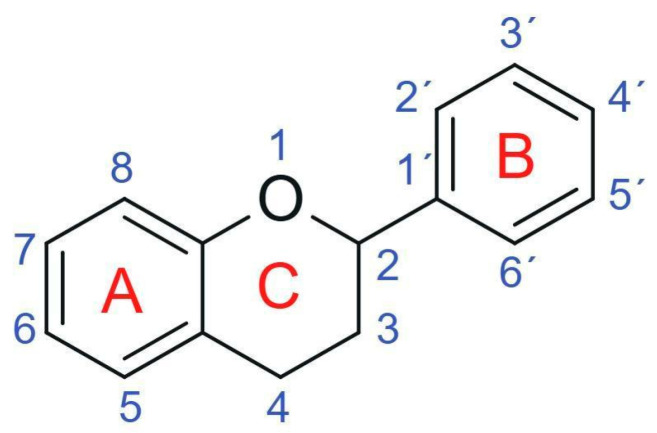
Basic structure of flavonoids. Author-generated illustration adapted from [2].

From a chemical perspective, flavonoids are polyphenolic compounds characterized by a basic C6-C3-C6 skeleton, consisting of two aromatic rings (A and B) linked by a three-carbon chain that forms a heterocyclic ring (C) [1], as illustrated in Figure 3.

The main flavonoid subclasses arise from variations in the degree of oxidation and saturation of the C ring, the position of attachment of the B ring, as well as the presence of the C2=C3 double bond and the carbonyl group at C4, along with different substitution patterns, including hydroxyl, methyl, methoxy, prenyl, and glycosyl groups, which directly influence reactivity, solubility, stability, and bioavailability [2,4,26].

The ubiquity of these metabolites in the human diet, from common fruits to medicinal teas, is a direct result of their diverse biosynthetic pathways in higher plants, where they constitute an important class of secondary metabolites and are found in fruits, vegetables, flowers, seeds, wine, and tea [1]. To date, more than 10,000 distinct flavonoids have been described and identified, reflecting their remarkable structural diversity derived from specific patterns of oxidation, hydroxylation, and glycosylation [27].

In plants, flavonoids perform essential functions, including regulating cell growth, attracting pollinating insects, and protecting against biotic and abiotic stresses. They also act as signaling molecules, ultraviolet radiation filters, and scavengers of reactive oxygen species [1,2]. The therapeutic versatility of these compounds, spanning from radical scavenging to complex immunomodulation, is detailed across various subclasses in Table 1 [1].

In plants, flavonoids may occur either in their free form (aglycones) or conjugated to sugar moieties, forming O- or C-glycosides, depending on the nature of the linkage between the sugar and the flavonoid core [4]. In general, O-glycosides predominate in plants, whereas C-glycosides and mixed O, C-glycosides are less frequent, although they have been reported in different plant species [28].

Although the different subclasses of flavonoids are widely distributed in natural sources and exhibit recognized biological relevance, their application in biological systems is mainly limited by chemical and biophysical properties, such as suboptimal aqueous dispersion, reduced chemical stability, restricted bioavailability, and unfavorable pharmacokinetics, in addition to constraints related to plant production and the processes of isolation and purification [29].

**Table 1:** Flavonoid subclasses: structural characteristics, examples, natural sources, and principal biological activities.

Basic Structure	Structural Differences	Representative Molecules	Natural Sources	Principal Activities	References
Flavones 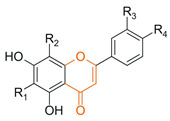	Double bond C2=C3 and carbonyl at C4	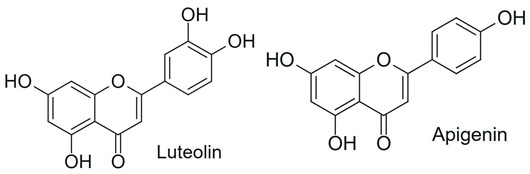	Citrus fruits, green tea, olive oil, broccoli, rosemary, oregano, parsley	Anti-inflammatory, anticancer, cardioprotective, antibacterial, antifungal, antiviral	[1,29,30]
Flavonols 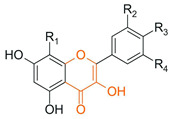	Flavone structure + hydroxyl group at C3	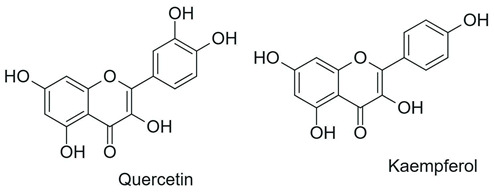	Onion, apple, kale, broccoli, grapes, tea	Anti-inflammatory, anticancer, cardioprotective, antibacterial, antifungal, antiviral	[1,29,30]
Flavanones 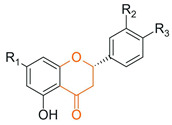	Saturation of the C2-C3 bond	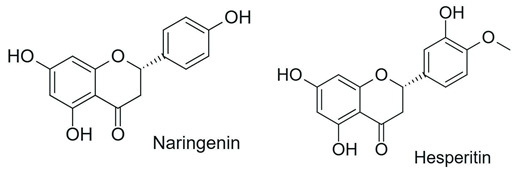	Citrus fruits (orange, lemon, grapefruit)	Anti-inflammatory, anticancer, cardioprotective, antifungal	[1,29,30]
Flavanols 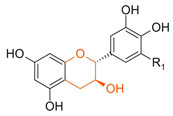	Absence of C2=C3 double bond and the carbonyl at C4; frequent presence of a hydroxyl group at C3	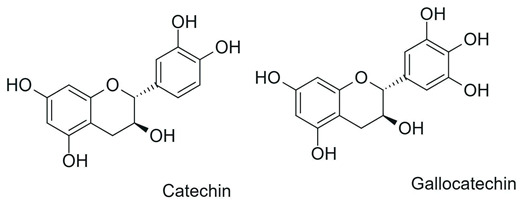	Green tea, cocoa, grapes, apple, red wine	Anticancer, antibacterial, antiviral	[1,29,30]
Anthocyanins 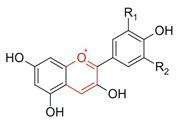	Presence of flavylium nucleus with a positive charge on the C ring associated with glycosylation	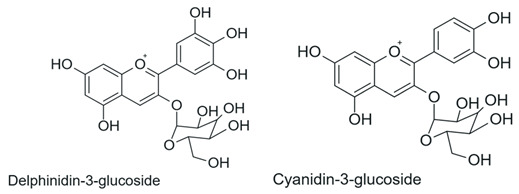	Red fruits (blackberry, blueberry, grapes), red cabbage	Anti-inflammatory and anticancer	[1,29,30]
Isoflavones 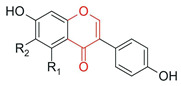	B ring attached at position C3 of the C ring	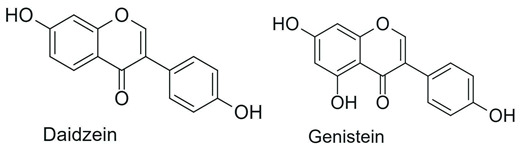	Soybean, chickpeas, beans, peanuts	Anticancer, antibacterial, antifungal, antiviral, cardioprotective	[1,29,30]
Chalcones 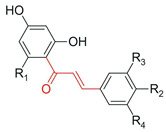	Open-chain structure, absence of the C ring (1,3-diaryl-2-propen-1-one)	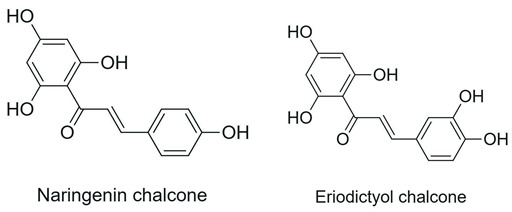	Soybean, apple, citrus fruits, ginger	Antioxidant, antibacterial, anthelmintic, antiulcerogenic, antiviral, antiprotozoal, anticancer	[2]

## 4. Bioavailability and Metabolism of Flavonoids

### 4.1. Absorption, Metabolism, and the Role of the Gut Microbiota

The challenge of achieving therapeutic plasma levels resides in the complex interplay between the flavonoid’s chemical form and the physiological barriers of the gastrointestinal tract [5,6]. In foods, flavonoids are predominantly ingested in glycoside form, which exhibits low intestinal permeability and therefore requires prior biotransformation steps to enable efficient absorption [4].

Following oral ingestion, only a fraction of flavonoids is absorbed in the upper gastrointestinal tract, while a significant proportion reaches the small intestine and colon, where it can interact with the intestinal microflora [5,6]. Structural characteristics and pH conditions modulate absorption, and once flavonoids cross the intestinal epithelium, they undergo processes such as oxidation, reduction, and dehydroxylation. Subsequently, metabolites are transported to the liver via the portal vein, where phase II reactions (conjugation) predominate, including glucuronidation, sulfation, and methylation, resulting in more polar and water-soluble compounds that facilitate urinary and biliary excretion [5].

As a consequence of the extensive metabolism occurring throughout the gastrointestinal tract and liver, flavonoids are largely converted into metabolites whose formation and biological activity are strongly modulated by the intestinal microbiota. These metabolites may exhibit biological effects distinct from those observed for the parent compounds evaluated in in vitro models, which helps explain the differences frequently observed between results obtained in cellular systems and in vivo responses [6].

**Figure 4:**
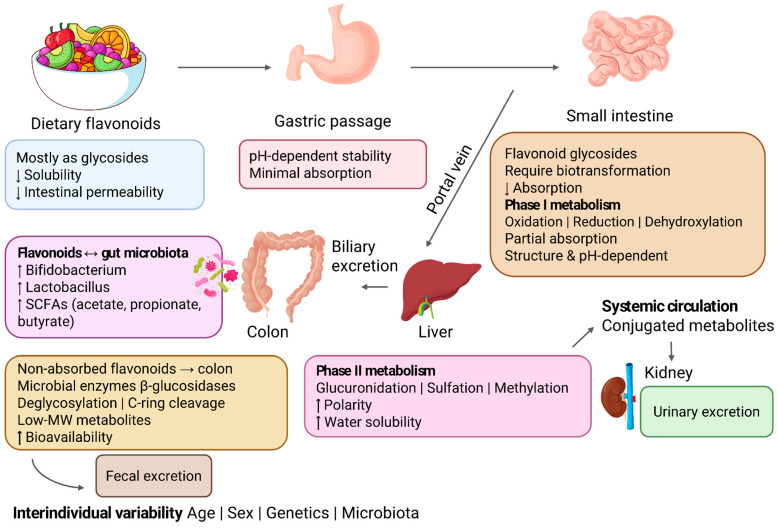
Schematic representation of flavonoid ADME (absorption, distribution, metabolism, and excretion). Author-generated illustration by Marília Crivelari da Cunha and Nicolly Clemente de Melo. Some graphical elements were created using resources from Freepik (https://www.freepik.com), accessed on 22 April 2026.

Figure 4 schematically illustrates the main stages of flavonoid pharmacokinetics, including absorption, distribution, metabolism, and excretion (ADME).

The gut microbiota plays a central role in the biotransformation of flavonoids that are not absorbed in the small intestine. Microbial enzymes, such as β-glycosidases and other hydrolases, catalyze reactions including deglycosylation, C-ring cleavage, demethylation, dehydroxylation, and reduction, generating phenolic metabolites with lower molecular weight that are often more bioavailable and biologically active [31].

In addition to being metabolized, flavonoids also exert modulatory effects on the composition and functional activity of the intestinal microflora. Evidence indicates that these compounds promote the growth of beneficial bacteria, such as species of the genera *Bifidobacterium* and *Lactobacillus*, while reducing the abundance of potentially pathogenic microorganisms. These changes are associated with increased production of short-chain fatty acids (SCFAs), such as acetate, propionate, and butyrate, which play a central role in maintaining intestinal barrier integrity, modulating immune responses, and regulating host energy metabolism [32].

The pharmacokinetics of flavonoids show high interindividual variability, influenced by factors such as sex, nutritional status, genetic profile, and intestinal microbiota composition. This variability affects the absorption, metabolism, and elimination of these compounds, making it difficult to standardize effective doses and to directly extrapolate experimental findings to clinical applications [5,33]. Furthermore, rapid urinary and biliary excretion, particularly observed for anthocyanins and flavonols, contributes to the short systemic residence time of these flavonoids [34].

In this context, it becomes evident that the low bioavailability and robust metabolism of flavonoids may represent critical obstacles to their therapeutic exploration. These challenges highlight the need for strategies capable of rationally modifying the structure of these compounds in order to improve their stability, absorption, and overall pharmacokinetic profile.

### 4.2. Strategies to Overcome Bioavailability Limitations

In view of the pharmacokinetic limitations discussed previously, several approaches have been proposed to enhance the bioavailability of flavonoids. Examples include nanoencapsulation systems and lipid-based carriers, which improve stability and intestinal absorption [35,36]; the formation of inclusion complexes with cyclodextrins and the development of solid dispersions, used to increase the aqueous solubility and stability of hydrophobic flavonoids [37]; the use of flavonoid transporter inhibitors to reduce phase II metabolism [38]; modulation of the food matrix, which directly influences intestinal absorption [39]; and modulation of the gut microbiome, capable of promoting structural modifications in flavonoids that favor their absorption and bioavailability [36].

The limitations affecting the therapeutic reach and reproducibility of flavonoid biological effects in vivo highlight the need for strategies capable of modifying their structural and metabolic characteristics. In this context, enzymatic modifications may be considered promising approaches with the potential to overcome absorption and metabolic barriers and, consequently, enhance the biological activity of these compounds [8,14].

## 5. Enzymatic Modifications of Flavonoids

### 5.1. Glycosylation and Deglycosylation

**Figure 5:**
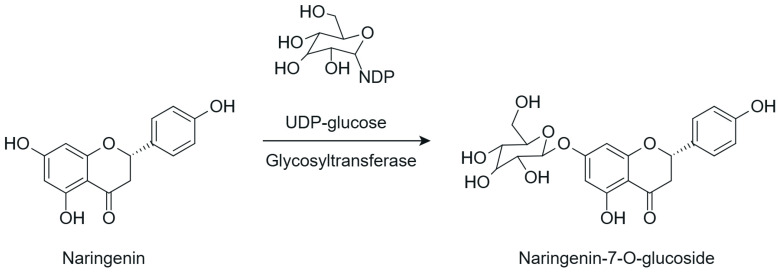
Glycosylation reaction of naringenin with UDP-glucose (NDP) catalyzed by glycosyltransferase. Author’s own illustration adapted [7].

Among the most commonly employed approaches, glycosylation stands out as a key modification, catalyzed by glycosyltransferases, particularly UDP-glycosyltransferases (UGTs). These enzymes transfer activated sugars, such as UDP-glucose, to hydroxyl groups of the flavonoid core, generating regioselective glycosides at positions such as 3-OH, 7-OH, and 4′-OH [40], as illustrated in Figure 5.

Recent studies further highlight that UGTs have been explored as tools for directed biocatalysis and metabolic engineering, enabling rational control over the position and type of sugar introduced, with a direct impact on the pharmacokinetic properties of flavonoids [41]. In this context, recent advances have enabled the functional characterization of flavonoid glycosyltransferases through heterologous expression systems, allowing detailed investigation of their substrate specificity and regioselectivity. For example, specific 5-O-glycosyltransferases involved in anthocyanin modification have been shown to catalyze the formation of stable 3,5-O-diglycosylated derivatives, contributing to flavonoid structural diversification and enhanced stability [42]. Similarly, recombinant glycosyltransferases exhibiting broad substrate scope and distinct positional selectivity have been reported, reinforcing their potential for controlled and targeted flavonoid modification [43].

Flavonoid glycosylation increases aqueous solubility and may enhance bioavailability, while generally reducing antioxidant activity and other in vitro bioactivities. However, in specific cases, it may generate derivatives with differentiated functions. The position of glycosylation and the nature of the conjugated sugar are key determinants of flavonoid ADME behavior, influencing intestinal absorption, metabolic stability, and systemic circulation time [8]. These factors may preserve or even enhance biological efficacy, despite reduced direct chemical reactivity [41].

Furthermore, the nature of the glycosidic bond significantly influences the metabolic fate of these compounds, since O-glycosides are more susceptible to hydrolysis, whereas C-glycosylated flavonoids exhibit greater structural stability and resistance to enzymatic action [4], features that are highly relevant for the rational adjustment of flavonoid pharmacokinetic properties.

Despite the broad interest in flavonoids and their glycosides, it remains difficult to establish general conclusions regarding the impact of glycosylation on bioactivity, as the effects depend on the type of flavonoid, its chemical structure, and the experimental context. Although O-glycosylation is frequently associated with reduced in vitro activity, glycosylated flavonoids may exhibit greater pharmacokinetic uptake and longer systemic circulation time in vivo, resulting in biological activity that is similar to or even greater than that of the corresponding aglycones. In contrast, the effects of C-glycosylation remain insufficiently explored, highlighting the need for additional physiological and clinical studies [44].

Recent advances have highlighted that enzymatic O-glycosylation represents an effective strategy to improve flavonoid bioavailability; however, achieving high regioselectivity remains a critical challenge, as it directly impacts product purity, catalytic efficiency, and the biological activity of the resulting derivatives [45].

**Figure 6:**
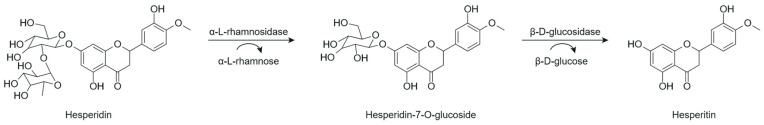
Scheme of the enzymatic deglycosylation of hesperidin through the sequential action of α-L-rhamnosidase and β-D-glucosidase, leading to the intermediate formation of hesperetin-7-O-glucoside and, subsequently, the aglycone hesperetin. Author-created image adapted [8].

On the other hand, the intentional deglycosylation of flavonoids, catalyzed by glycosidases, mainly α-L-rhamnosidases and β-D-glycosidases, promotes the hydrolysis of glycosidic bonds and the release of aglycones from glycosylated flavonoids (Figure 6). These enzymes may act sequentially and, in industrial contexts, are often employed in multicomponent enzymatic preparations, such as naringinase and hesperidinase, which are widely used as biocatalytic tools [8].

Although deglycosylation also occurs naturally during digestion, mediated by intestinal enzymes and microbiota, these processes differ from controlled enzymatic approaches used in technological applications. In this applied context, enzymatic deglycosylation systems have been explored across different industrial sectors, particularly for increasing the fraction of flavonoids in their aglycone form, reducing the bitterness of citrus juices, and enhancing aroma in wines, without relying on in vivo metabolic transformations [8,46].

### 5.2. Acylation

**Figure 7:**
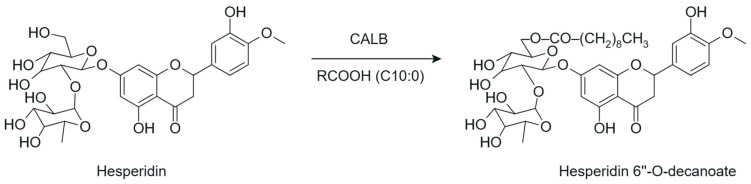
Scheme of the enzymatic acylation of hesperidin by CALB, resulting in the formation of the monoacylated derivative 6”-O-decanoyl hesperidin from the fatty acid C10:0. Author’s own illustration adapted [9].

Another relevant strategy for structural modification is acylation, carried out through esterification or transesterification reactions catalyzed by microbial lipases, particularly *Candida antarctica* lipase B (CALB/Novozym 435) and *Thermomyces lanuginosus* lipase (TLL/Lipozyme^®^ TL IM), widely studied and frequently used in their immobilized forms due to their high selectivity and efficiency under mild reaction conditions [47]. This modification promotes the controlled introduction of acyl chains into the flavonoid structure (Figure 7), resulting in targeted changes in the hydrophilic-lipophilic balance and in their physicochemical properties [9,48].

The introduction of acyl groups reduces the polarity associated with the presence of multiple hydroxyl groups, promoting increased lipophilicity, compatibility with lipid systems, and the chemical stability of flavonoids, particularly in oily matrices [48]. In this context, the use of natural triglycerides as acyl donors enables the production of flavonoid esters that are structurally more stable and better suited for incorporation into technological systems [49].

From a structure-function perspective, enzymatic acylation enables the rational adjustment of flavonoid lipophilicity; however, this modification must be carefully controlled, as excessive increases in lipophilicity may compromise the aqueous solubility of the derivatives. Recent evidence also indicates that acyl-glycosylated derivatives, particularly those bearing acyl chains of intermediate length, exhibit an optimized balance between lipophilicity and hydrophilicity, highlighting the potential of enzymatic acylation as a strategy for targeted structural modification [13].

### 5.3. Hydroxylation

**Figure 8:**
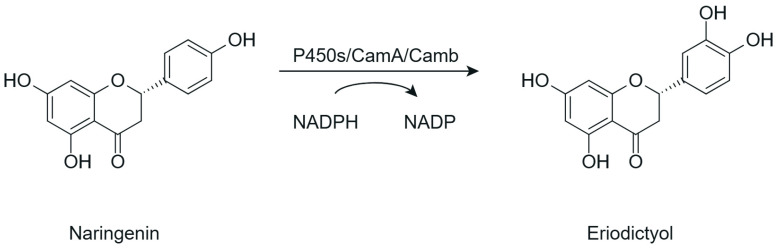
Hydroxylation of naringenin to eriodictyol catalyzed by NADPH-dependent bacterial cytochrome P450, with the participation of the redox proteins CamA and CamB. Author’s image adapted [50].

Another relevant strategy for the structural modification of flavonoids is hydroxylation, which enables the selective introduction of hydroxyl groups at specific positions of the flavonoid core. This modification is primarily catalyzed by cytochrome P450-dependent monooxygenases or flavin-dependent monooxygenases, which are widely distributed in microorganisms and plants and have been explored as biocatalytic tools for promoting site-specific hydroxylation under physiologically compatible conditions [50]. A representative example of regioselective flavonoid hydroxylation catalyzed by cytochrome P450-dependent monooxygenases is illustrated in Figure 8.

The additional introduction of hydroxyl groups modifies properties such as polarity, hydrogen-bonding capacity, and electronic reactivity of the flavonoid core, linking the structural diversification promoted by enzymatic hydroxylation to possible differences in the behavior of these compounds in biological and technological systems [50,51].

In plant systems, hydroxylases such as flavonoid 3′-hydroxylase (F3′H) and flavonoid 3′,5′-hydroxylase (F3′5′H) play a central role in defining hydroxylation patterns of the B ring, directing the formation of flavonoids with different degrees and positions of hydroxyl substitution and contributing to the structural diversity observed in natural metabolites [52].

### 5.4. Methylation

Flavonoid methylation is a natural biological process catalyzed by S-adenosyl-L-methionine (SAM)-dependent O-methyltransferases (OMTs), in which methyl groups are transferred to phenolic hydroxyls of the flavonoid core, generating methoxylated derivatives with distinct regioselective patterns. Although this reaction occurs naturally in the secondary metabolism of plants and microorganisms, in the biotechnological context, it has been intentionally explored by cloning and heterologously expressing genes encoding OMTs, thereby enabling rational control of flavonoid structural modification [53].

Methylation can occur via the oxygen or carbon atom, resulting, respectively, in O-methylated or C-methylated compounds; however, O-methylation is the form most frequently explored in enzymatic strategies, due to the greater accessibility of phenolic hydroxyls and the high regioselectivity exhibited by O-methyltransferases [54].

Within this group, flavonoid O-methyltransferases (FOMTs) stand out, as they exhibit high regioselectivity and can act at different hydroxylated positions, such as 3′-, 5′-, and 7-OH, in addition to accepting a wide variety of flavonoids with different structural skeletons as substrates. This catalytic versatility favors the directed diversification of methoxylated products, as demonstrated in studies based on heterologous expression and microbial biotransformation [11,53].

**Figure 9:**
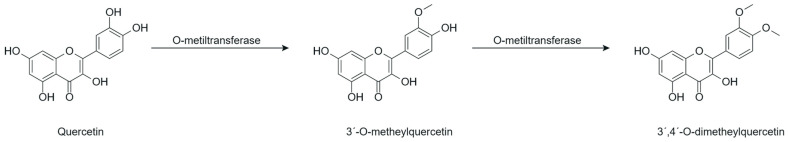
O-methylation of quercetin catalyzed by O-methyltransferases, leading to the formation of mono- and dimethylated derivatives. Author’s image adapted [55].

Figure 9 illustrates a typical enzymatic O-methylation reaction, highlighting the regioselective introduction of methoxy groups into the flavonoid core.

In citrus, highly regioselective O-methyltransferases promote the formation of polymethoxylated flavones (PMFs) with specific methylation patterns, revealing the molecular basis of their structural diversity. Methylation reduces the polarity of phenolic hydroxyl groups, thereby altering the hydrophilic–lipophilic balance, protecting reactive sites, and enhancing chemical and metabolic stability, membrane permeability, and pharmacokinetic properties compared with non-methylated forms. When applied through recombinant systems, this controlled enzymatic modification exhibits high catalytic selectivity, with regioselectivity depending on both the enzyme and the substrate [11,56].

Recent studies have demonstrated the potential of enzymatic methylation as a strategy to generate flavonoid derivatives with distinct properties. In this context, O-methyltransferases from *Citrus depressa* (CdMT5) and *Sorghum vulgare* (SvMT) were heterologously expressed and employed to catalyze the conversion of quercetin into eight methoxylated derivatives via biotransformation, which were subsequently isolated, identified, and obtained with high purity (>95%). In addition, these compounds were evaluated in terms of stability, cellular uptake, and effects on lipid metabolism using a 3T3-L1 cell model [57]. The results demonstrated that methylation significantly enhanced chemical stability, whereas cellular uptake and biological effects varied depending on the substitution pattern. Derivatives such as 5,7,3′,4′-tetramethylquercetin and rhamnetin significantly reduced intracellular triglyceride levels, while other compounds promoted adipogenesis, highlighting a structure–activity relationship dependent on both the position and degree of methoxylation. In particular, the retention of the C3–OH group and dual methoxylation of the A-ring were identified as key structural features influencing biological activity. These findings indicate that enzymatic methylation not only improves physicochemical properties but also directly modulates the biological effects of flavonoid derivatives, reinforcing the importance of structural control in the development of compounds with specific activities.

### 5.5. Prenylation

**Figure 10:**
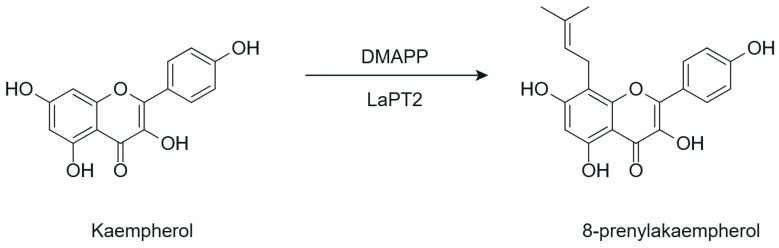
Scheme of kaempferol prenylation mediated by the prenyltransferase LaPT2 (*Lupinus albus*), with DMAPP (dimethylallyl pyrophosphate) as the prenyl donor, leading to the formation of 8-prenylkaempferol. Author’s image adapted [58].

Prenylation constitutes a relevant strategy for the structural modification of flavonoids, widely recognized as a biosynthetic mechanism capable of expanding the chemical diversity of these metabolites. This process is catalyzed by prenyltransferases (PTs), which promote the incorporation of prenyl groups at specific positions of the flavonoid core, allowing the controlled production of prenylated derivatives from precursor flavonoids [58,59]. The general scheme of this enzymatic transformation is presented in Figure 10.

Although prenylated flavonoids are widely described in phytochemical studies, recent interest has focused on prenylation as an enzymatic tool for the directed transformation of flavonoids. In this context, PTs exhibit distinct catalytic behaviors that vary in origin, specificity, and biotechnological applicability. Plant PTs, predominantly belonging to the UbiA superfamily, are often membrane-associated and tend to exhibit higher substrate specificity, which poses challenges for heterologous expression and limits their use in recombinant systems [60].

In contrast, PTs of microbial origin have been explored as more accessible alternatives for the enzymatic transformation of flavonoids, since they generally exhibit higher solubility, easier heterologous expression, and, in some cases, broad substrate specificity, favoring their application in metabolic engineering strategies and the directed synthesis of prenylated flavonoids under controlled conditions [12].

From a structural perspective, the introduction of prenyl groups promotes an increase in the lipophilicity of flavonoids, altering their affinity for hydrophobic environments and facilitating their interaction with and penetration into biological membranes [59,61]. These physicochemical modifications expand the repertoire of derivatives accessible through enzymatic chemical modification strategies.

### 5.6. Enzymes and Microbial Systems: Implications and Biotechnological Applications

The technological application of flavonoid biotransformation reactions is directly related to the availability of biocatalysts and the strategies adopted for their production, which may involve either isolated enzymes, which allow greater experimental control and the precise adjustment of reaction parameters, such as pH, temperature, solvent type and enzyme/substrate ratio, favoring the development of more predictable processes and the standardization of experimental protocols [7].

In this context, enzyme immobilization has been widely employed as a strategy to increase the operational stability of biocatalysts, enabling their reuse, facilitating process control, and contributing to cost reduction in industrial applications [62]. In addition, the high chemo-, regio-, and stereoselectivity of enzymes favors the production of products with a high degree of purity, frequently eliminating the need for additional purification steps and positively impacting the overall yield of the processes [7]. Approaches based on genetic and protein engineering, such as directed evolution and rational design, have been explored to improve the performance of these biocatalysts, expanding their stability, catalytic efficiency, and robustness under more demanding reaction conditions [63].

In contrast, whole microbial systems provide a cellular environment favorable for maintaining enzymatic conformation and for cofactor regeneration; however, their practical application is often limited by intrinsic metabolic complexity, the need to select strains with suitable catalytic performance, and the sensitivity of microorganisms to organic solvents, factors that may compromise cellular integrity and reduce catalytic efficiency. Moreover, the use of wild-type microorganisms, such as species of *Aspergillus and Penicillium*, may result in metabolic variability, the formation of undesired by-products, and challenges related to standardization and scalability [7,64].

From a practical perspective, among the most commonly used commercial biocatalysts are glycosidases (*naringinase and hesperidinase)*, widely employed in the deglycosylation of flavonoids, as well as *Candida antarctica* lipase B (CALB/Novozym 435) and *Thermomyces lanuginosus* lipase B (Lipozyme TL IM), applied in regioselective acylation reactions that expand the structural diversity of the obtained derivatives. In contrast, modifications such as enzymatic prenylation and methylation remain largely dependent on recombinant microbial systems, since the enzymes involved are not yet widely available in commercial formulations [11,60].

Thus, the combination of enzymatic biocatalysis, recombinant microbial systems, metabolic engineering, and process optimization constitutes a complementary and promising strategy for the sustainable development of flavonoid derivatives with pharmaceutical and nutraceutical potential.

Despite these advances, challenges related to large-scale production, enzyme stability, and cost-effectiveness remain significant barriers to the industrial and pharmaceutical application of enzymatically modified flavonoids [7,63].

## 6. Evaluation of the Therapeutic Properties of Enzymatically Modified Flavonoids

### 6.1. Antioxidant and Antimicrobial Properties

In general, enzymatic modifications exert distinct effects on the antioxidant and antimicrobial activity of flavonoids, as they directly alter functional groups involved in the scavenging of reactive species, in the interaction with biological membranes, and in the modulation of molecular targets. These effects depend on the type of modification introduced, the structural position affected, and the experimental model employed, highlighting the importance of distinguishing between in vitro, in vivo, and cellular evidence when interpreting these outcomes.

Glycosylation is often associated with reduced antioxidant capacity in phenolic compounds, particularly in chemical radical-scavenging assays, as the attachment of sugar moieties may block phenolic hydroxyl groups essential for electron or hydrogen donation and free radical stabilization. This effect is structure-dependent and influenced by the flavonoid class, as well as the number, position, and type of conjugated sugar [8,65]. Experimental evidence of this behavior has been reported for citrus flavonoid glycosides, where the formation of mono- and polyglycosides of compounds such as naringin, neohesperidin, neodiosmin, and rutin resulted in unchanged or reduced antioxidant activity in DPPH and FRAP assays, while increasing solubility in aqueous media. However, these findings are primarily based on in vitro assays, which may not fully reflect biological activity under physiological conditions. Additionally, variations in flavonoid structure, glycosylation patterns, and assay conditions may contribute to inconsistencies across studies [16].

From an antimicrobial perspective, glycosylation also tends to reduce the activity of flavonoids compared with their aglycone forms. This effect is attributed to increased polarity and reduced lipophilicity, which impair the amphipathic balance required for interaction with microbial membranes, thereby limiting membrane insertion, permeabilization, and access to intracellular targets [8,66,67]. Furthermore, these antimicrobial effects are mostly derived from in vitro models, and their relevance in complex biological systems remains to be further validated.

On the other hand, the deglycosylation of flavonoids is frequently associated with enhanced antioxidant capacity, as it can release phenolic hydroxyl groups that participate in electron or hydrogen donation and restore key structural features, such as electronic conjugation and molecular planarity. However, these effects are not universal and depend on the flavonoid structure, the position of the glycosidic linkage, and the experimental conditions. When present, such structural changes may promote greater stabilization of the resulting flavonoid radical and improve efficiency in radical-scavenging and reducing processes [68,69]. Experimental evidence indicates that the conversion of glycosylated flavonoids into aglycones is often associated with increased antioxidant activity. In guava leaves, fermentation and enzymatic hydrolysis promoted the conversion of glycosides into aglycones, resulting in significant increases in antioxidant activity in DPPH, ABTS, NO_2_^−^, and reducing power assays [70]. Similarly, the enzymatic hydrolysis of flavanones from orange peel catalyzed by immobilized naringinase promoted the formation of aglycones such as prunin and naringenin, accompanied by increased antioxidant activity in the DPPH assay [71]. However, in flavonoids from chamomile aqueous infusion, partial deglycosylation did not significantly alter antioxidant activity, indicating that the effect depends on the matrix and flavonoid class [72]. Regarding antimicrobial activity, deglycosylation generally increases antimicrobial potency due to increased lipophilicity and improved interaction with microbial membranes and intracellular targets [73,74]. However, the magnitude of these effects varies considerably depending on the flavonoid class, the extent of hydrolysis, and the experimental model employed, which may limit the generalization of these findings.

Acylation modulates antioxidant activity, primarily by influencing the distribution of flavonoids between aqueous and lipid phases. The introduction of acyl chains increases the affinity of the derivatives for hydrophobic environments, favoring their activity in lipid systems and in assays based on hydrophobic radicals, such as DPPH. From a biochemical perspective, these derivatives tend to position themselves more efficiently at lipid interfaces, where they can interrupt chain reactions of lipid peroxidation. However, the relationship between increased lipophilicity and antioxidant activity is not linear, and the so-called cutoff effect is often observed, in which excessively long acyl chains increase steric hindrance and reduce antioxidant efficiency [75]. In addition, acylation has been associated with increased antimicrobial activity of flavonoids, an effect mainly attributed to the increased lipophilicity of the derivatives, which favors their interaction with microbial membranes, leading to increased permeability and structural disruption of the lipid bilayer, particularly for derivatives containing acyl chains of intermediate length [76,77]. Nevertheless, the relationship between lipophilicity and biological activity is complex, and excessive hydrophobicity may reduce bioavailability and limit effectiveness in biological systems.

Hydroxylation of flavonoids constitutes a structural modification capable of promoting relevant changes in their biological properties by introducing new hydroxyl groups at specific positions of the molecule. Variants of the monooxygenase CYP450 BM3 have been shown to catalyze the regioselective hydroxylation of flavonoids, such as the conversion of naringenin into eriodictyol through hydroxylation at the C-3′ position of the B ring. Although antioxidant activity was not directly evaluated, the hydroxylated derivatives obtained showed higher antimicrobial activity against Gram-positive bacteria. This effect was associated with a greater capacity to interact with microbial cellular components, possibly involving hydrogen bonding with nucleic acids and interference in essential processes such as DNA and RNA synthesis. However, the lack of direct evaluation of antioxidant activity in some studies limits the interpretation of the full impact of hydroxylation on redox-related properties [78].

Methylation of flavonoids, catalyzed by S-adenosyl-L-methionine-dependent methyltransferases, can selectively modulate different biological activities. Some O- and C-methylated flavonoids maintain or exhibit antioxidant activity and may display cytoprotective effects in cellular systems. In addition, O-methylated flavonoids may exhibit antimicrobial activity, generally associated with effects on membrane integrity and microbial metabolic processes [54]. Regioselective methylation through enzymatic biotransformation has also been shown to increase antimicrobial activity against bacteria and fungi, with the position of the methoxy group on the flavonoid skeleton playing an important role in biological activity [19]. However, these effects are highly dependent on the position of methylation and the biological system evaluated, which may lead to variable outcomes across different studies.

Prenylation modulates the antioxidant and antimicrobial activities of flavonoids by increasing their affinity for lipid environments and their interaction with membranes. In aqueous systems, prenylation tends to maintain or only moderately enhance radical-scavenging activity, and may reduce efficacy when increased lipophilicity compromises solubility [59]; however, prenylated flavonoids show good antioxidant performance in lipid systems, such as in the inhibition of lipid peroxidation and LDL oxidation [61]. In the antimicrobial context, prenylation is associated with increased antibacterial and antifungal activity, including bactericidal effects, antibiofilm activity, and synergism with antibiotics, reinforcing its role as a relevant structural modification [61,79]. Additionally, increased lipophilicity may limit solubility in aqueous environments, potentially reducing effectiveness in certain biological contexts.

Overall, the biological effects of enzymatic modifications on flavonoids are highly context-dependent and influenced by structural, physicochemical, and experimental factors, highlighting the need for integrated studies that combine in vitro, in vivo, and clinical approaches.

Table 2 summarizes selected experimental studies investigating the effects of enzymatic modifications on the antioxidant and antimicrobial activities of flavonoids in in vitro, cellular, and in vivo models.

**Table 2:** Selected studies on enzymatic modifications of flavonoids and their antioxidant and antimicrobial effects.

Type of Modification	Flavonoid/Substrate	Obtained Derivative(s)	Enzyme/Strategy	Model	Results Obtained	**References**
Glycosylation	Rutin	α-monoglucosyl rutin	Cyclodextrin glucanotransferase (CGTase)	In vivo (mice)	↓ oxidative stress; ↑ hepatic antioxidant activity	[80]
Glycosylation	Naringin, neohesperidin, neodiosmin, rutin	Monoglycosides and polyglycosides of citrus flavonoids	α-L-rhamnosidase + CGTase	In vitro (DPPH, FRAP)	~ antioxidant activity (model-dependent); ↑ solubility	[16]
Deglycosylation	Flavanones from orange peel albedo (naringin, narirutin, hesperidin, neohesperidin, rutin)	Prunin and naringenin	Naringinase (α-L-rhamnosidase + β-D-glucosidase), immobilized on corn cob	In vitro (DPPH)	↑ antioxidant activity (DPPH) after enzymatic hydrolysis	[71]
Deglycosylation	Flavonoids from the aqueous infusion of chamomile flowers (*Matricaria chamomilla* L.)	Aglycones and partially deglycosylated flavonoids	Hesperidinase + β-galactosidase	In vitro (DPPH)	↔ antioxidant activity	[72]
Deglycosylation	Flavonoids from guava leaf infusion (*Psidium guajava* L.)	Aglycones	Fermentation + enzymes (cellulase, xylanase, hemicellulase, and β-glucosidase)	In vitro (DPPH, ABTS, NO_2_^−^ scavenging and reducing power)	↑ DPPH scavenging; ↑ ABTS^+^ scavenging; ↑ NO_2_^−^ scavenging; ↑ reducing power	[70]
Glycosylation and acylation	Dihydromyricetin (DMY)	DMY glycosides and acylglycosylated DMY derivatives	Mutant phosphorylase and TLL (Lipozyme TL IM)	In vitro (DPPH, ABTS)	↓ antioxidant activity after glycosylation; acylation attenuates the loss (ABTS > DPPH)	[81]
Glycosylation and acylation	Phloretin	Acylated phloretin α-glycoside (C8, C12, C16)	Mutant phosphorylase R134A and TLL (Lipozyme TL IM)	In vitro (DPPH, ABTS)	↓ slight antioxidant activity in DPPH; ↔ activity in ABTS	[13]
Acylation	Naringin	6″-O-(3-hydroxybutyryl) naringin	CALB (Novozym 435); TLL (Lipozyme TL IM); RML (Lipozyme RM IM)	In vitro (DPPH, ABTS)	↓ antioxidant activity (DPPH); ↓ antioxidant activity (ABTS)	[82]
Acylation	Naringin	Naringin acetate, propionate, and laurate	CALB (Novozym 435)	In vitro (DPPH, β-carotene/linoleic acid assay)	↓ antioxidant activity in DPPH; ↑ lipid protection in the β-carotene assay (laurate > propionate > acetate)	[76]
Acylation	Flavonoids from bamboo leaves	Acylated bamboo leaf flavonoids	CALB (Novozym 435)	In vitro (DPPH, FRAP, β-carotene/linoleic acid)	↓ slight activity in DPPH; ↓ activity in FRAP; ↑ antioxidant activity in lipid systems (β-carotene)	[83]
Acylation	Rutin	Acylated rutin derivatives	TLL (Lipozyme TL IM)	In vitro (DPPH, Fe^2+^ chelation, β-carotene–linoleate assay)	↓ DPPH scavenging; ↔ Fe^2+^ chelation; ↑ inhibition of lipid peroxidation (β-carotene-linoleate)	[84]
Acylation	Baicalin	Baicalin esters	CALB (Novozym 435)	In vitro (MIC: *Staphylococcus aureus* ATCC 6538; *Escherichia coli* GIM 1.707; *Candida albicans* ATCC 10231	↑ antimicrobial activity; cutoff effect (C log P ~5.2); membrane disruption (*S. aureus*, *C. albicans*)	[77]
Acylation	Blackcurrant Anthocyanins (*Ribes nigrum* L.)	Monoacylated anthocyanins with lauric acid.	CALB (Novozym 435)	In vitro (DPPH; β-carotene/linoleic acid)	↓ activity in aqueous medium (DPPH); ↑ protection in lipid system (β-carotene)	[85]
Acylation	Cyanidin-3-O-galactoside (cy-gal) from Alpine Bearberry (*Arctostaphylos alpina* L.)	Monoacylated cy-gal (C12, C14, C16, C18)	CALB (Novozym 435)	In vitro (DPPH, FRAP)	↔ antioxidant activity (DPPH); ↔ reducing power (FRAP)	[86]
Hydroxylation	Naringenin	Eriodictyol	CYP450 BM3 variant M13 (biocatalysis; in vitro and whole-cell)	In vitro (DD assay: *Bacillus subtilis*, *Micrococcus luteus*, *Staphylococcus aureus*, *Pseudomonas aeruginosa*, and *Enterobacter cloacae*)	↑ antibacterial activity against Gram-positive bacteria (*M. luteus* > *S. aureus* ≈ *B. subtilis*)	[78]
Methylation	Liquiritigenin, Naringenin, and Hesperidin	O-methylated flavonoids	O-methyltransferase (HsOMT, LtOMT) expressed in *S. cerevisiae*	In vitro (MIC/MBC; *Staphylococcus aureus ATCC* 6538; *Escherichia coli ATCC* 25922; *Candida albicans* SC5314)	↑ antimicrobial activity (*C. albicans*, *S. aureus*, *E. coli*)	[19]

Legend: ↑ significant increase (*p* < 0.05); ↓ significant decrease (*p* < 0.05); ↔ no statistically significant difference (*p* ≥ 0.05), when reported in the original study. Effects are presented as described by the respective authors; ~ trend toward reduction without statistical significance. In vitro: assays performed in chemical systems or cell cultures; In vivo: assays performed in animal models. DPPH: 2,2-diphenyl-1-picrylhydrazyl; ABTS: 2,2′-azino-bis(3-ethylbenzothiazoline-6-sulfonic); FRAP: ferric reducing antioxidant power; β-carotene/linoleic acid: lipid protection assay; NO_2_^−^ scavenging: reactive nitrogen species scavenging assay; DD assay: disk diffusion assay. MIC: minimum inhibitory concentration; MBC: minimum bacterial concentration. CGTase: cyclodextrin glucanotransferase; CALB: *Candida antarctica* lipase B; TLL: *Thermomyces lanuginosus* lipase; RML: *Rhizomucor miehei* lipase; CYP450 (P450): cytochrome P450; whole-cell: biocatalysis in whole cells. C log P: calculated partition coefficient, indicative of lipophilicity (cutoff effect).

### 6.2. Anti-Inflammatory Properties

Inflammation is a complex biological response triggered by foreign agents or tissue damage, involving the activation of immune receptors and signaling cascades that lead to the production of cytokines, chemokines, and other inflammatory mediators, as well as the recruitment of immune cells such as macrophages, lymphocytes, and neutrophils [27,87].

When the inflammatory stimulus is not efficiently eliminated, the response may become chronic, characterized by the persistent activation of pro-inflammatory mediators. Among the main pathways involved in this process are Toll-like receptors (TLRs), the mitogen-activated protein kinase (MAPK) pathway, and the nuclear factor kappa B (NF-kB) pathway, which is responsible for regulating the expression of pro-inflammatory cytokines and enzymes such as cyclooxygenase-2 (COX-2) and inducible nitric oxide synthase (iNOS) [87].

The activation of these pathways promotes the release of inflammatory mediators, such as prostaglandins, leukotrienes, nitric oxide, and reactive oxygen species, contributing to the amplification of the inflammatory response, as well as to the development of pain, edema, and tissue damage. The dysregulation of these mechanisms is associated with several inflammatory and degenerative diseases, including cardiovascular diseases, diabetes, arthritis, Alzheimer’s disease, asthma, and cancer [27,87].

In this context, flavonoids have been widely investigated due to their ability to interfere with multiple molecular targets involved in inflammation. These compounds can modulate pro-inflammatory pathways, reduce the production of cytokines and inflammatory mediators, and attenuate oxidative stress associated with inflammation, contributing to the prevention and control of exacerbated inflammatory processes [27,87].

Studies indicate that glycosylated flavonoids may exert anti-inflammatory effects by modulating key signaling pathways, including NF-κB, MAPK, and STAT3, thereby reducing the release of inflammatory mediators and attenuating tissue damage. Experimental evidence demonstrates that glycosylated derivatives of quercetin, catechins, and kaempferol can reduce the secretion of pro-inflammatory cytokines such as TNF-α and IL-6 [41]. However, these effects are highly dependent on the flavonoid structure, the glycosylation pattern, and the experimental model employed, and do not necessarily indicate superior activity compared with their aglycone forms.

In addition to the modulation of classical inflammatory pathways, flavonoids have been shown to influence cellular homeostasis through mechanisms related to mitochondrial function and metabolic regulation. In this context, cyanidin-3-O-glucoside (C3G), a naturally occurring anthocyanin flavonoid, has been shown to promote PINK1/Parkin-mediated mitophagy, leading to reduced oxidative stress, suppression of NLRP3 inflammasome activation, and attenuation of hepatic steatosis in experimental models of non-alcoholic fatty liver disease [88]. Although this compound is not enzymatically modified, glycosylated flavonoids such as C3G can also be obtained through enzymatic processes. These findings provide important mechanistic insights into the biological effects of flavonoids and support the rationale that enzymatic modification strategies may enhance their pharmacokinetic properties and therapeutic efficacy. In addition, anthocyanins such as C3G have been widely associated with beneficial effects on metabolic disorders, including type 2 diabetes, through anti-inflammatory activity, glycemic control, and modulation of gene expression [89]. These observations reinforce the need for further investigation in clinical settings.

Similarly, acylation of flavonoids has been associated with enhanced anti-inflammatory activity in various experimental models. Acetylated, p-coumaroylated, and galloylated derivatives have been shown to reduce inflammatory edema, inhibit nitric oxide and prostaglandin production, and suppress pro-inflammatory cytokines such as TNF-α, IL-1β, and IL-6, in some cases exhibiting activity comparable to or greater than reference drugs like indomethacin. These findings highlight acylation as a relevant structural strategy for modulating inflammatory responses, with potential application to enzymatically modified flavonoids, depending on the acyl group and its position [90]. However, these effects may vary depending on the acyl group and its position, as well as the experimental model employed.

Methylation also influences anti-inflammatory activity, with effects strongly dependent on the position of the methyl group and the mediator evaluated. Evidence indicates that specific methylation patterns may enhance the inhibition of prostaglandin E_2_ (PGE_2_) and nitric oxide, while having a limited impact on cytokines such as IL-8 or TNF-α [30], and may lead to variable outcomes depending on the biological system and target evaluated. However, these findings are mainly derived from previous literature, as studies directly evaluating the anti-inflammatory effects of enzymatically methylated flavonoids remain limited among the studies included in this review.

Despite the growing evidence regarding glycosylated and acylated flavonoids, studies evaluating the anti-inflammatory effects of other enzymatic modifications, such as methylation, hydroxylation, and prenylation, remain limited, highlighting an important gap in the literature and the need for further systematic investigation. Overall, although enzymatic modifications can significantly modulate the biological activity of flavonoids, most available evidence is derived from in vitro and in vivo studies. Therefore, the translation of these findings to clinical applications should be interpreted with caution, and well-designed clinical studies are still required to confirm the therapeutic relevance of these modified compounds. The selected studies addressing the anti-inflammatory effects of enzymatically modified flavonoids are summarized in Table 3.

### 6.3. Antitumor and Antiproliferative Activities

Flavonoids have been widely studied for their ability to modulate oxidative stress and cellular metabolism, exhibiting antitumor and antiproliferative activities. Cancer is a heterogeneous disease characterized by uncontrolled cell proliferation, dysregulation of the cell cycle, resistance to apoptosis, chronic inflammation, angiogenesis, and metastatic potential, and is associated with oxidative stress, genetic mutations, and mitochondrial dysfunction. In this context, flavonoids exert anticancer effects by regulating reactive oxygen species (ROS) homeostasis, inducing cell cycle arrest, activating apoptotic pathways, and suppressing the proliferation and invasiveness of tumor cells, acting either as antioxidants or as pro-oxidants under pathological conditions [91].

Systematic studies indicate that flavonoid glycosides tend to exhibit lower antiproliferative and antitumor activity in vitro models when compared with their aglycone forms. Both O- and C-glycosylation may reduce the ability of these compounds to inhibit cell proliferation, an effect mainly attributed to increased hydrophilicity and steric hindrance, which limit their penetration across cellular membranes and their interaction with intracellular targets [30].

In general, O-glycosylation of flavonoids is associated with reduced antiproliferative and antitumor activity compared with aglycones; however, this effect is not unidirectional [44]. The masking of phenolic hydroxyl groups is frequently associated with decreased biological activity, which may reduce interactions with biological targets [8]. For example, isorhamnetin-3-O-rhamnoside exhibited moderate and cell line-dependent antiproliferative effects, with reduced proliferation observed in MCF-7 cells but not in HepG2 or A549 cells [92]. Nevertheless, there is evidence that structurally modified glycosylated flavonoids, such as glycosylated biflavonoids, may display antiproliferative activity equal to or greater than that of their aglycone counterparts due to more favorable physicochemical properties, including increased solubility, selectivity, and cellular interaction [93].

**Table 3:** Selected studies on enzymatic modifications of flavonoids and their anti-inflammatory effects.

Type of Modification	Flavonoid/Substrate	Obtained Derivative(s)	Enzyme/Strategy	Model	Observed Results	References
Glycosylation	Rutin	α-monoglucosyl rutin	Cyclodextrin glucanotransferase (CGTase)	In vivo (mice with cyclophosphamide-induced liver injury)	↓ ALT; ↓ AST; ↓ TBA; ↓ LPS hepatic; ↓ IL-6; ↓ IL-1β; ↓ TNF-α; ↓ inflammatory infiltration; ↑ antioxidant defense	[80]
Glycosylation	Naringin, Neohesperidin, Rutin	Monoglucosides and Polyglycosides of citrus flavonoids	α-L-rhamnosidase + CGTase	In vitro (RAW 264.7 macrophages stimulated with LPS)	↓ NO production; ↔ cell viability (>90%); effect dependent on glucose position/number and C-ring structure	[16]
Acylation	Naringin	6″-O-(3-hydroxybutyryl) naringin	CALB (Novozym 435); TLL (Lipozyme TL IM); RML (Lipozyme RM IM)	In vitro (murine BMDCs stimulated with LPS)	~ TNF-α; ↓ IL-10 (3HBN > naringin)	[82]
Acylation	Quercetin-3-O-glucoside	Quercetin-3-O-glucoside–EPA ester	CALB (Novozym 435)	In vitro (human THP-1-derived macrophages stimulated with LPS); In vivo (Wistar rats with a high-fat diet and LPS-induced inflammation)	↓ TNF-α; ↓ IL-6; ↓ IFN-γ; ↓ COX-2; ↓ PGE_2_; ↓ NF-κB; ↓ CRP; ↑ adiponectin; ~ TNF-α (in vivo); ↔ IL-10	[17]
Acylation	Phloridzin (PZ)	PZ-DHA (DHA ester)	CALB (Novozym 435)	In vitro (human THP-1-derived macrophages stimulated with LPS)	↓ TNF-α; ↓ IL-6; ↓ COX-2; ↓ PGE_2_; ↓ nuclear translocation of NF-κB; ↔ cell viability	[18]

Legend: ↑ significant increase (*p* < 0.05); ↓ significant decrease (*p* < 0.05); ↔ no statistically significant difference (*p* ≥ 0.05), when reported in the original study. Effects are presented as described by the respective authors; ~ trend toward reduction without statistical significance. Experimental models: In vitro: assays performed in cell cultures; In vivo: assays performed in animal models. Biochemical and inflammatory markers: ALT: alanine aminotransferase; AST: aspartate aminotransferase; TBA: total bile acids; LPS: lipopolysaccharide; NO: nitric oxide; TNF-α, IL-6, IL-1β, IL-10, IFN-γ: inflammatory cytokines; COX-2: cyclooxygenase-2; PGE_2_: prostaglandin E_2_; NF-κB: nuclear factor kappa-B; CRP: C-reactive protein; Adiponectin: anti-inflammatory adipokine. Cell lines and cellular models: RAW 264.7: murine macrophages; BMDCs: bone marrow–derived dendritic cells; THP-1: human monocytes differentiated into macrophages. Fatty acids: DHA: docosahexaenoic (22:6 n-3).

Deglycosylation can reduce cellular proliferation in vitro, as observed in the products of partial rutin hydrolysis by hesperidinase [94] and in the hydrolysis of flavonones from orange peel albedo catalyzed by naringinase [71]. In general, the presence of sugar moieties in flavonoids reduces their interaction with key molecular targets. It attenuates their direct antitumor efficacy in cell culture, whereas deglycosylation exposes aromatic hydroxyl groups and restores electronic conjugation, potentially increasing cytotoxicity and antiproliferative activity in vitro [91]. The aglycones formed may induce cell-cycle arrest, activate pro-oxidant pathways, and modulate signaling pathways, including PI3K/Akt/mTOR and HIF-1α. They may also promote apoptosis and autophagy, representing a recurrent pattern of higher cellular potency compared with glycosides [95]. However, despite the increased in vitro activity, aglycones often exhibit lower solubility and faster metabolism in vivo. In contrast, glycosides may act as transport forms or prodrugs, being deglycosylated by intestinal enzymes or gut microbiota to release the active aglycones [96]. Thus, although deglycosylation generally favors direct antiproliferative activity in cellular systems, the overall antitumor effect is not unidirectional and depends on the flavonoid structure, the type of sugar moiety, and the biological context. Exceptions exist in which certain glycosides maintain, or even exceed, the activity of their corresponding aglycones [93,96,97].

Taken together, these findings indicate that the effects of glycosylation and deglycosylation on in vivo antitumor activity remain insufficiently understood and require further investigation, particularly considering that most available evidence is derived from in vitro studies that do not fully capture the complexity of metabolism, bioavailability, and interactions with the intestinal microbiota.

Acylation is a relevant chemical modification capable of modulating the biological properties of flavonoids. Generally, acylation tends to increase lipophilicity and membrane permeability, favoring cellular interaction and, in many cases, enhancing antitumor and antiproliferative activities. In glycosylated flavonoids, acylation of sugar moieties often intensifies bioactivity by enhancing membrane permeation and interactions, whereas direct acylation of aglycone hydroxyl groups may reduce classical antioxidant capacity but favor inhibition of tumor cell proliferation and anti-inflammatory effects [98]. Acylated derivatives of rutin, obtained through the introduction of benzoate groups catalyzed by *Thermomyces lanuginosus* lipase, showed a significant reduction in cell proliferation in tumor cell lines, with a more pronounced effect in MCF-7 cells and low cytotoxicity in normal cells [84]. Similarly, acylation of quercetin-3-O-glycoside with long-chain fatty acids (C12–C18), mediated by *Candida antarctica* lipase, resulted in strong inhibition of cell proliferation in HepG2 cells, associated with cell cycle arrest in the S phase and the induction of apoptosis, while maintaining low toxicity toward normal hepatic cells [20].

In the case of hydroxylation, this structural modification may be associated with enhanced anticancer properties of flavonoids, in a manner strongly dependent on the substitution pattern. In most cases, the introduction of hydroxyl groups at key structural positions, particularly in the B ring, leading to the formation of a catechol motif (3′,4′-dihydroxylated), has been associated with a greater ability to inhibit cell proliferation, modulate the cell cycle, and induce apoptosis in tumor cells [99]. This effect is associated with the influence of hydroxylation pattern on the redox activity of flavonoids, which determines their ability to scavenge free radicals, chelate metal ions, and modulate reactive oxygen species. These properties enable both classical antioxidant activity and a controlled pro-oxidant behavior in tumor cells [91,95,100]. However, this effect is not uniform, since the number and position of hydroxyl groups directly influence bioactivity, and excessive increases in hydrophilicity may limit interactions with cellular targets. Therefore, hydroxylation contributes to the enhancement of anticancer activity only when it occurs at structurally favorable positions [30].

Methylation represents a structural modification capable of modulating the antiproliferative and anticancer activity of flavonoids. In general, O-methylation increases lipophilicity and metabolic stability, favoring cellular penetration, access to intracellular targets, and interaction with proteins involved in the regulation of cell death [30]. When occurring at structurally favorable positions, such as 3′ or 4′ of the B ring, methylation has been associated with greater antiproliferative and cytotoxic effects in different tumor cell lines. These effects include the induction of apoptosis through caspase activation, mitochondrial damage, and modulation of p53, as well as the suppression of pro-tumorigenic pathways related to inflammation and cell survival, such as NF-κB and PARP-1, in addition to the inhibition of enzymes involved in carcinogenesis, including CYP1A1/1B1 and aromatase [30,101]. On the other hand, methylation at less favorable positions, such as substitution of the hydroxyl group at position 5 of the A ring, may significantly reduce anticancer activity [30]. Thus, the impact of methylation is not uniform, as the final effect depends on the substitution pattern and the flavonoid backbone, with a balanced combination of methoxy and hydroxyl groups being crucial to optimize lipophilicity, redox activity, and tumor selectivity [30,101].

Prenylation of flavonoids, involving the introduction of isoprenyl or geranyl chains, is generally associated with enhanced antitumor and antiproliferative activities, primarily due to increased lipophilicity, which improves membrane interaction and facilitates access to intracellular molecular targets [59,61]. Prenylated flavonoids often exhibit higher bioactivity and selective cytotoxicity against different tumor cell lines compared with their non-prenylated analogs, while maintaining low toxicity toward normal cells, with the prenyl group frequently playing a key role in antiproliferative potency [59,102]. Evidence from structure–activity relationship studies indicates that the number and position of prenyl chains play a critical role in bioactivity, with particular relevance for prenylation at positions such as C-3, C-6, C-8, or C-3′, as well as for diprenylated flavonoids, which tend to exhibit greater cytotoxicity than their monoprenylated counterparts [59,61,102,103]. Mechanistically, prenylation facilitates flavonoid entry into cells and promotes the activation of pathways associated with cell death, including cell cycle arrest and caspase activation, in addition to interfering with oncogenic signaling axes and inflammatory processes [59,61]. However, these effects remain highly dependent on the substitution pattern, the flavonoid scaffold, and the experimental model.

Overall, enzymatic modifications can substantially influence the biological activity of flavonoids; however, most of the available evidence is based on in vitro and in vivo studies. Therefore, caution is required when extrapolating these findings to clinical contexts, and additional well-designed human studies are necessary to confirm their therapeutic relevance.

The selected studies addressing enzymatic modifications of flavonoids and their antitumor and antiproliferative effects are summarized in Table 4.

**Table 4:** Selected studies on enzymatic modifications of flavonoids and their antitumor and antiproliferative effects.

Type of Modification	Flavonoid/Substrate	Obtained Derivative(s)	Enzyme/Strategy	Model	Observed Results	References
Glycosylation	Biflavonoids (structurally diverse biflavonoid aglycones)	Biflavonoid monoglycosides (1a, 2a, 4a, 5a) and diglycosides (1b, 3b)	O-glycosyltransferase UGT74AN2 coupled with sucrose synthase AtSuSy (UDP-glucose regeneration)	In vitro (PC-3 cells)	↑ water solubility (20–980×); ↑ antiproliferative activity for compound 1a vs. aglycone (↓ IC_50_); ~ activity for other glycosylated derivatives (structure-dependent)	[93]
Glycosylation	Vitexin	β-D-fructofuranosyl-(2→6)-vitexin; β-D-difructofuranosyl-(2→6)-vitexin	β-fructosidase from *Arthrobacter nicotianae*	In vitro (MCF-7 and MDA-MB-231 cells)	↓ cell viability; ↑ antitumor activity compared to vitexin; reduced IC_50_ for both derivatives; similar effect between mono- and difructosylation	[104]
Glycosylation	Isorhamnetin	Isorhamnetin-3-O-rhamnoside	Enzymatic cascade (rhamnosyltransferase 78D1 from *A. thaliana*, sucrose synthase, and UDP-rhamnose synthase, with UDP-rhamnose regeneration)	In vitro (HepG2, MCF-7 and A549 cells)	↓ cell proliferation in MCF-7; moderate antiproliferative effect dependent on cell line.	[92]
Deglycosylation	Flavones from orange peel albedo (naringin, narirutin, hesperidin, neohesperidin, and rutin)	Prunin and naringenin	Naringinase (α-L-rhamnosidase + β-D-glucosidase), immobilized on corn cob.	In vitro (SW480 cells)	↓ cell viability in SW480 cells; antiproliferative effect dependent on concentration.	[71]
Deglycosylation	Rutin	Quercetin-3-O-glucoside (Q3G/isoquercitrin) and quercetin	Hesperidinase (*Penicillium* sp.; α-L-rhamnosidase and β-D-glucosidase activities)	In vitro (CHO-K1 cells)	↓ cell proliferation in CHO-K1; absence of mutagenicity; ↑ antimutagenic effect dependent on Q3G content.	[94]
Deglycosylation	*Sophora japonica* extract is rich in kaempferol glycosides	Kaempferol-enriched extract (KPF-ABR)	Hesperidinase + β-galactosidase	In vitro (NG-97 and U251 cells; HDFa as non-tumoral control)	↓ cell viability and proliferation; induction of apoptosis; cell cycle arrest; ↓ migration/invasion; inhibition of MMP-9/NF-κB; stronger effect of biotransformed extract (KPF-ABR)	[105]
Acylation	Rutin	Rutin acyl derivatives with benzoic acid (2′′′-, 4′′′-e 2′′-O-benzoates)	Lipase TLL (Lipozyme TL IM)	In vitro (HepG2, Caco-2, MCF-7 cells; LO-2 as normal control)	↓ cell proliferation; ↑ anticancer activity after acylation; stronger effect in MCF-7; compound 2 with lower EC_50_; low cytotoxicity in LO-2	[84]
Acylation	Quercetin-3-O-glycoside	Long chain acylated esters of quercetin-3-O-glucoside (C12–C18)	Lipase CALB (Novozym 435)	In vitro (HepG2 cells)	↓ cell proliferation (≈85–90%); cell cycle arrest at S phase; induction of apoptosis; low toxicity in normal hepatic cells	[20]
Hydroxylation	Naringenin	Eriodictyol	CYP450 BM3 variant M13 (biocatalysis; in vitro and whole-cell)	In vitro (AGS, HCT116, HepG2 and HeLa cells)	↓ cell viability; higher anticancer potential of eriodictyol compared to naringenin.	[78]
Methylation	Liquiritigenin, naringenin, and hesperidin	O-methylated flavonoids	O-methyltransferases (HsOMT, LtOMT) expressed in *S. cerevisiae*	In vitro (MCF-7 cells)	↓ cell proliferation; ↑ antiproliferative potency (IC_50_ ≈ 10.31 μM); low relative cytotoxicity.	[19]
Methylation	Hydroxylated flavonoids (mainly flavones and flavonols with vicinal hydroxyls)	O-methylated flavonoids	O-methyltransferase CrOMT2 (SAM as methyl donor)	In vitro (SGC-7901 and BGC-823 cells)	↓ cell viability; ↑ cytotoxicity of methylated flavonoids, especially after 3′methylation in compounds with C2–C3 double bond	[15]
Prenylation	Dimethylallylated flavonoids (precursors of icariin)	Prenylated icariin mimetics (O- and C-prenylated)	Prenyltransferase AtaPT (donors: DMAPP, GPP, FPP)	In vitro (5637 cells)	↓ cell viability; selective cytotoxic; IC_50_ in the range of 14–20 μM; low cytotoxicity in normal cells	[106]

Legend: ↑ significant increase (*p* < 0.05); ↓ significant decrease (*p* < 0.05), when reported in the original study. Effects are presented as described by the respective authors; ~ trend toward reduction without statistical significance. In vitro: assays performed in cell cultures. Whole-cell: biocatalysis in whole cells, using living cells as enzymatic systems with endogenous cofactor regeneration. IC_50_: half-maximal inhibitory concentration required to reduce 50% of cell viability; EC_50_: effective concentration required to reduce 50% of cell proliferation. Enzymes and cofactors: OMT: O-methyltransferase; HsOMT: O-methyltransferase from *Hordeum vulgare*; LtOMT: O-methyltransferase from *Lotus tenuis*; CrOMT2: O-methyltransferase from *Citrus reticulata*; CALB: *Candida antarctica* lipase B; TLL: *Thermomyces lanuginosus* lipase (Lipozyme TL IM); CYP450 (P450): cytochrome P450, a family of monooxygenases involved in regioselective oxidative reactions; SAM: S-adenosyl-L-methionine; DMAPP: dimethylallyl diphosphate; GPP: geranyl diphosphate; FPP: farnesyl diphosphate. Compounds: Q3G: quercetin-3-O-glucoside (isoquercitrin). Cell lines: MCF-7: human breast adenocarcinoma; MDA-MB-231: human triple-negative breast carcinoma; HepG2/HepG2: human hepatocellular carcinoma; Caco-2: human colorectal carcinoma; A549: human lung carcinoma; AGS: human gastric adenocarcinoma; HCT116: human colorectal carcinoma; HeLa: human cervical carcinoma; PC-3, human prostate adenocarcinoma (androgen-independent); SGC-7901 and BGC-823: human gastric cancer cell lines; SW480: human colorectal adenocarcinoma; 5637: human bladder carcinoma; CHO-K1: Chinese hamster ovary cell line; NG-97 and U251: human glioma cell lines; LO-2: normal human hepatic cell line; HDFa: normal human dermal fibroblasts. Other markers: MMP-9: matrix metalloproteinase 9; NF-κB: nuclear factor kappa B.

### 6.4. Clinical Evidence of Flavonoid Derivatives: Implications for Cancer and Other Diseases

Data from the PubChem database were used to map flavonoid–disease associations. Based on the LIPID MAPS classification system (Polyketides [PK12]), 6048 flavonoids were identified. The “Associated Diseases and Disorders” section was accessed using Python scripts to retrieve compounds with reported disease associations. Among these, only 142 flavonoids were associated with diseases, comprising 75 distinct conditions, including six cancer types.

The diseases most frequently associated with flavonoids were endometrial carcinoma (7.8%), vascular disease (5.9%), bone disease (5.5%), nephrotic syndrome (4.6%), and primary ovarian insufficiency (4.6%). These findings indicate that flavonoid–disease associations predominantly involve the vascular, renal, skeletal, and endocrine systems, which aligns with the well-established physiological and pharmacological roles of flavonoids in these areas.

**Figure 11:**
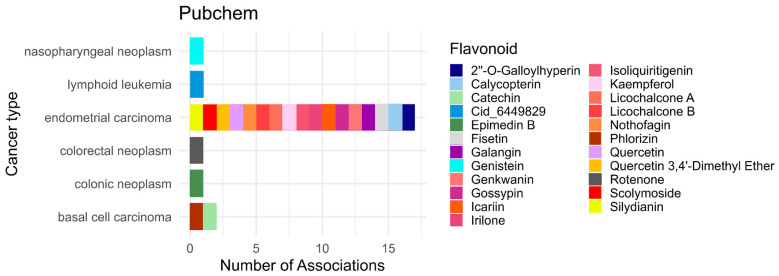
PubChem data illustrating cancer types reported to be associated with different flavonoids.

Among these conditions, endometrial carcinoma emerged as the most recurrent disease, appearing in 17 distinct records. Other types of neoplasms were also identified, including colorectal neoplasms, basal cell carcinoma, lymphoid leukemia, and nasopharyngeal neoplasms, albeit with lower frequency (Figure 11). Oncological diseases were prominently represented among the identified associations, underscoring the growing scientific interest in investigating flavonoids as compounds with potential antitumor activity.

The compounds most frequently associated with neoplastic diseases include quercetin, kaempferol, fisetin, galangin, icariin, and licochalcone A (well-characterized bioactive flavonoids). In experimental models, these molecules have demonstrated the ability to inhibit tumor growth, induce apoptosis, and modulate signaling pathways involved in cancer progression. The recurrent associations between these compounds and diseases such as endometrial carcinoma may therefore reflect not only their pharmacological relevance but also the accumulated scientific evidence supporting their biological activity.

Conversely, some isolated associations (such as those involving rotenone or other less-studied compounds) may be related to toxic or context-dependent effects, as the PubChem database integrates information from studies of various natures, including pharmacological, toxicological, and observational data. Hence, the presence of an association does not necessarily imply a therapeutic effect or causal relationship, but rather a correlation documented in the scientific literature.

**Figure 12:**
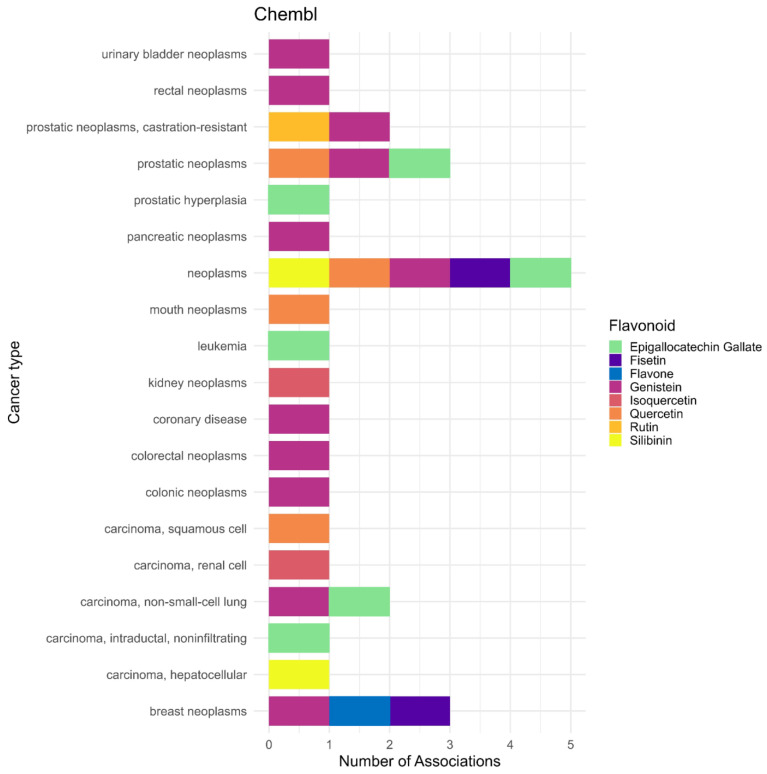
ChEMBL database: cancer types in which flavonoids have been investigated in clinical trials.

Clinical trials conducted over the last decade have evaluated natural flavonoids and other polyphenols as potential therapeutic agents in oncology, due to their ability to modulate cellular pathways related to tumor proliferation, cell cycle regulation, and the induction of apoptosis (Figure 12). In this context, a systematic review of phase II and III clinical trials evaluated the use of natural flavonoids as chemotherapeutic agents in different types of cancer. The studies included patients with hematopoietic and lymphoid neoplasms as well as solid tumors, and flavonoids were predominantly administered in pharmacological formulations, often in combination with conventional chemotherapy regimens. More consistent clinical responses were observed in hematopoietic and lymphoid tumors, whereas the results in solid tumors were generally limited. The authors also highlighted considerable heterogeneity among protocols, doses, routes of administration, and experimental designs as a factor that complicates the consolidation of robust clinical conclusions. It is important to note that the studies analyzed did not involve flavonoids obtained through enzymatic structural modification [107].

The COSMOS randomized clinical trial, conducted with more than 21,000 adults aged 60 years or older, evaluated supplementation with a flavanol-rich cocoa extract (500 mg/day). Although no statistically significant reduction was observed in the primary endpoint of total cardiovascular events, an approximate 27% reduction in cardiovascular mortality was reported in the supplemented group compared with the placebo group, suggesting a potential benefit for specific outcomes [108].

In contrast, clinical data on enzymatically modified flavonoids remain scarce, particularly in the oncological context. To date, there is no clinical evidence supporting the therapeutic application of these derivatives in cancer treatment, highlighting an important gap in the literature and reinforcing the need for well-designed clinical investigations evaluating the safety, bioavailability, and efficacy of these compounds. Nevertheless, some studies in humans have evaluated enzyme-modified flavonoids in other clinical contexts, indicating the potential of these strategies to improve specific functional properties. In general, these modifications involve enzymatic glycosylation reactions, in which glucose residues are added to the flavonoid molecule, resulting in derivatives with higher water solubility and potentially improved absorption in the gastrointestinal tract.

In this context, enzymatically modified isoquercitrin (EMIQ), an oligoglycosylated derivative obtained through quercetin glycosylation, has been investigated due to its high water solubility and increased bioavailability. In a clinical trial conducted with athletes, supplementation with EMIQ combined with whey protein increased muscle mass and improved antioxidant status, suggesting that greater systemic availability of quercetin metabolites may contribute to exercise-related physiological adaptations [109]. Additionally, acute administration of EMIQ in individuals at cardiovascular risk increased the pharmacokinetic uptake of circulating quercetin metabolites and improved endothelial function, as evidenced by increased flow-mediated dilation [110]. These findings support the hypothesis that enzymatic modification can optimize the absorption and metabolism of flavonoids, enhancing their beneficial vascular effects.

Monoglucosyl rutin, produced by enzymatic transfer of glucose to rutin, has been investigated due to its increased solubility and digestibility. In a randomized, double-blind, placebo-controlled clinical trial, ingestion of this compound for 8 weeks resulted in a significant reduction in visceral abdominal fat, indicating a potential metabolic effect of the modified flavonoid [111]. Subsequent studies from the same research group demonstrated additional beneficial effects, including attenuation of postprandial glycemic elevation, improvement of vascular flexibility, and reduction in LDL-cholesterol levels in healthy adults, reinforcing the cardiometabolic potential of these glycosylated derivatives.

The same authors also demonstrated that a single dose of monoglucosyl rutin was able to significantly suppress postprandial glycemic elevation in healthy adults with relatively elevated fasting glucose levels after the ingestion of a carbohydrate-rich meal, without the occurrence of adverse effects, suggesting that enzymatic glycosylation may improve the functional properties of rutin and contribute to postprandial glycemic control [112].

In more recent studies, the same research group specifically evaluated the impact of monoglucosyl rutin on lipid profile, demonstrating that daily supplementation for 12 weeks promoted significant reductions in serum LDL-cholesterol levels, as well as total and non-HDL cholesterol, in adults with mild hypercholesterolemia, without observed adverse effects. These findings reinforce the role of monoglucosyl rutin as a safe functional ingredient with potential preventive effects in reducing cardiovascular risk [113].

In a subsequent study by the same group, the effects of daily intake of monoglucosyl hesperidin, either alone or in combination with monoglucosyl rutin, were evaluated in healthy adults with low vascular flexibility. The combination of the two glycosylated flavonoids promoted significant improvement in endothelial function, evidenced by increased flow-mediated dilation, reduced arterial stiffness, and decreased inflammatory markers compared with placebo. These findings indicate that the coadministration of glycosylated polyphenols with greater solubility and bioavailability may exert a synergistic effect in improving vascular health in individuals with cardiometabolic risk [114].

Despite promising bioactivity reported in experimental models, the limited number of clinical studies on enzymatically modified flavonoids may be attributed to multiple factors, including regulatory challenges, high costs associated with enzyme production and process scalability, and significant interindividual variability in flavonoid metabolism driven by genetic background and gut microbiota composition [5,33,63].

## 7. Conclusions

Enzymatic modification of flavonoids has emerged as a robust and versatile strategy to expand structural diversity and fine-tune key physicochemical and biological properties, including solubility, stability, bioavailability, and target-specific activity. Approaches such as glycosylation, methylation, acylation, hydroxylation, and prenylation enable controlled and regioselective structural changes, allowing the generation of derivatives with distinct pharmacokinetic profiles and biological effects. Evidence from preclinical studies demonstrates that these modifications can significantly alter absorption, metabolic stability, and cellular responses, highlighting the potential of enzymatically modified flavonoids in the context of oxidative stress–related and chronic diseases, including cardiovascular disorders, inflammation, metabolic diseases, and cancer. However, most of the available evidence is still derived from in vitro and animal models, while well-designed human studies remain limited, and the high heterogeneity among studies, particularly regarding precursor compounds, enzymatic systems, modification conditions, and experimental models, restricts direct comparisons and the establishment of consistent structure–activity relationships. In addition, the limited integration of biological outcomes with pharmacokinetic data, metabolism, and safety remains a critical barrier to translation, especially considering that enzymatic modifications may involve trade-offs between chemical reactivity and bioavailability. Future advances will depend on methodological standardization, deeper investigation of structure–activity and structure–metabolism relationships, and the integration of biocatalysis with emerging tools such as metabolic engineering and computational design, alongside the expansion of well-designed in vivo and clinical studies. Overall, enzymatic modification represents a powerful platform for the rational design of flavonoid derivatives with optimized and predictable properties, with strong potential to bridge the gap between promising preclinical findings and real-world applications in pharmaceutical, nutraceutical, and functional food systems.

## Data Availability

The data used in this study were obtained from publicly available databases (PubChem and ChEMBL). The processed data are available from the corresponding author upon reasonable request.

## Supplementary Materials

The following supporting information can be downloaded at: https://www.mdpi.com/article/10.3390/antiox15050539/s1, Supplementary File S1: PRISMA 2020 checklist; Supplementary File S2: Search Strategy for all databases.

## Abbreviations

ADME	Absorption, Distribution, Metabolism, and Excretion
ABTS	2,2′-Azino-bis(3-ethylbenzothiazoline-6-sulfonic acid)
ALT	Alanine aminotransferase
AST	Aspartate aminotransferase
BMDCs	Bone marrow–derived dendritic cells
CALB	Candida antarctica lipase B
CGTase	Cyclodextrin glucanotransferase
ChEMBL	Chemical Database of Bioactive Molecules with Drug-Like Properties
COX-2	Cyclooxygenase-2
CRP	C-reactive protein
CYP450	Cytochrome P450
DD assay	Disk diffusion assay
DMAPP	Dimethylallyl diphosphate
DPPH	2,2-Diphenyl-1-picrylhydrazyl
EC_50_	Half-maximal effective concentration
FPP	Farnesyl diphosphate
FRAP	Ferric reducing antioxidant power
GPP	Geranyl diphosphate
IC_50_	Half-maximal inhibitory concentration
IFN-γ	Interferon gamma
IL	Interleukin
LPS	Lipopolysaccharide
MAPK	Mitogen-activated protein kinase
MBC	Minimum bactericidal concentration
MIC	Minimum inhibitory concentration
NF-κB	Nuclear factor kappa B
NO	Nitric oxide
OMT	O-methyltransferase
PMFs	Polymethoxylated flavones
PRISMA	Preferred Reporting Items for Systematic Reviews and Meta-Analyses
PTs	Prenyltransferases
ROS	Reactive oxygen species
SAM	S-adenosyl-L-methionine
SCFAs	Short-chain fatty acids
STAT3	Signal transducer and activator of transcription 3
TBA	Total bile acids
TLL	Thermomyces lanuginosus lipase
TNF-α	Tumor necrosis factor alpha
UGTs	UDP-glycosyltransferases
